# Trends in suicide death risk in transgender people: results from the Amsterdam Cohort of Gender Dysphoria study (1972–2017)

**DOI:** 10.1111/acps.13164

**Published:** 2020-03-12

**Authors:** C. M. Wiepjes, M. den Heijer, M. A. Bremmer, N. M. Nota, C. J. M. de Blok, B. J. G. Coumou, T. D. Steensma

**Affiliations:** ^1^ Department of Endocrinology Amsterdam UMC VU University Medical Center Amsterdam the Netherlands; ^2^ Center of Expertise on Gender Dysphoria Amsterdam UMC VU University Medical Center Amsterdam the Netherlands; ^3^ Department of Psychiatry Amsterdam UMC VU University Medical Center Amsterdam the Netherlands; ^4^ Department of Medical Psychology Amsterdam UMC VU University Medical Center Amsterdam the Netherlands

**Keywords:** gender dysphoria, transgender, suicide

## Abstract

**Objective:**

This study explored the overall suicide death rate, the incidence over time, and the stage in transition where suicide deaths were observed in transgender people.

**Methods:**

A chart study, including all 8263 referrals to our clinic since 1972. Information on death occurrence, time, and cause of death was obtained from multiple sources.

**Results:**

Out of 5107 trans women (median age at first visit 28 years, median follow‐up time 10 years) and 3156 trans men (median age at first visit 20 years, median follow‐up time 5 years), 41 trans women and 8 trans men died by suicide. In trans women, suicide deaths decreased over time, while it did not change in trans men. Of all suicide deaths, 14 people were no longer in treatment, 35 were in treatment in the previous two years. The mean number of suicides in the years 2013–2017 was higher in the trans population compared with the Dutch population.

**Conclusions:**

We observed no increase in suicide death risk over time and even a decrease in suicide death risk in trans women. However, the suicide risk in transgender people is higher than in the general population and seems to occur during every stage of transitioning. It is important to have specific attention for suicide risk in the counseling of this population and in providing suicide prevention programs.

**Significant outcomes**

- Suicide death risk in trans people did not increase over time.
- Suicide deaths occurred during every stage of transitioning.
- Suicide death risk is higher in trans people than in the general population.

**Limitations**

- Psychological comorbidity was not known.
- No data were available for people on the waiting list for their first appointment.

## Introduction

Gender dysphoria (GD) refers to the distress related to a marked incongruence between one’s assigned gender at birth and the experienced gender (1). Trans people are diverse in the intensity of experienced GD (2), their needs for medical transition (3), and the impairment that GD can have on their life. Studies focusing on the wellbeing of trans people show a greater vulnerability for experiencing mental health problems compared with the non‐trans (cis) population (4). Most prevalent are affective and anxiety problems (5, 6, 7, often accompanied by feelings, thoughts, or behaviours linked to suicidality (8, 9.

The prevalence of suicidality in trans people in suicidal ideation, suicidal attempts, and suicide death rates is studied in varying degrees and shows high variability in findings. A systematic review by McNeil et al (9). reported suicidal ideation rates across 17 identified studies, ranging from 37% (10) up to 83% (11). Prevalence rates on suicidal attempts in trans people, which are generally observed to be lower than suicidal ideation, showed to be lower but also with a wide variation in reported rates, ranging from 9.8% (12) up to 44% (13). Since structured prevalence studies on suicide deaths are lacking in the transgender literature, an estimation comes from a limited number of studies reporting on suicide death rates in small study samples. Derived from a systematic review on suicidality in trans people by Marshall et al. (8), suicide death rates varied from 0% (14) to 4.2% in a sample of 24 post‐treatment trans people from Sweden (15). Six of these studies only included postsurgical people (14, 15, 16, 17, 18, 19, whereas two studies also included trans people who were only using hormones without surgery (20, 21. However, studies differentiating the treatment stage during which death by suicide occurred are lacking. In addition, studies differentiating between suicide in trans women and trans men are scarce. While some studies found that trans men have a higher risk of suicide attempts than trans women (22, 23, other studies reported no differences in suicide attempts between trans women and trans men (24, 25. Only one cohort distinguished suicide death risk in trans women and trans men and found that trans women had an increased risk of suicide death compared with trans men (20, 21.

### Aims of the study

The aim of the current study is to explore the overall suicide death rate in trans women and trans men in the largest clinical cohort of gender‐referred people seen at the Center of Expertise on Gender Dysphoria of the Amsterdam University Medical Centers between 1972 and 2017 the Netherlands (26). In addition, the change in incidence of suicide death rate over time and at what stage in transition (pretreatment, during hormonal treatment and/or surgical phase, or post‐treatment) suicide deaths were observed was explored. The relevance of such information is to get a greater understanding of how large the risk is in clinically referred transgender people and whether suicide prevention interventions should focus on specific stages in transition or not.

## Material and methods

### Study design

A retrospective chart study was performed, including all people who once visited the Center of Expertise on Gender Dysphoria of the Amsterdam UMC, Vrije Universiteit Amsterdam, the Netherlands, between 1972 and 2017. The selection of the study population is described previously (26). A total of 8263 adults, adolescents, and children were included, with a median age at first visit of 25 years (range 4 to 81 years) and a median follow‐up time of 7.5 years (range 0.0 to 45.5 years). Information on death occurrence, time, and cause of death was obtained by cross‐checking multiple sources: the National Civil Record Registry (21), which contains date of birth and date of death of all inhabitants of the Netherlands, and the hospital registration system, medical, and psychological files for cause of death.

The Medical Ethics Review Committee of the Amsterdam UMC, Vrije Universiteit Amsterdam, reviewed this study and determined that the Medical Research Involving Human Subjects Act (WMO) did not apply to this study. Therefore, and because of the retrospective design, necessity for informed consent was waived.

### Treatment

After an initial visit to the endocrinologist (for adults) or child psychiatrist (for children and adolescents), all people were referred to the psychology department for the diagnostic phase. In this phase, people were seen to gain insight into their experienced gender identity, to verify whether they fulfill the diagnosis gender dysphoria, to explore their treatment desires, and to prepare them for possible medical interventions. After this phase, people may start with hormonal treatment. Trans women received treatment with anti‐androgens and estrogens. Trans men were treated with testosterone. In adolescents, treatment first started with a period of puberty suppression, followed by estrogens of testosterone around the age of 16 years (27).

Surgical interventions can be offered to people aged 18 years or older. Depending on the desired treatment, the surgery is preceded after at least one year of hormonal treatment (genital surgery) or can be offered after the diagnostic phase (e.g., breast removal). After surgery, all people were usually seen every 2 years for medical check‐up.

### Statistical analyses

Characteristics of the population were shown as median with range due to the non‐normal distribution. The total number of people seen at our center and the total number of suicide deaths were counted and were expressed as percentages as well as incidence per 100 000 person years. For each year, the number of people at risk and the number of people who died by suicide were calculated. Cox regression analyses were performed to calculate hazard ratios (HR) with corresponding 95% confidence intervals (95% CI). Date of first visit was used as start date of follow‐up. The end date of follow‐up was either date of death or date of closing the database (December 31, 2017). Suicide death was analyzed as event. To analyze whether the incidence of suicide deaths changed over time, the year of first visit was added as determinant to the analyses. Analyses were adjusted for age at first visit as age might be related to suicide death risk. Time between date of suicide death and first visit, and between date of suicide death and start of hormonal treatment, if applicable, were calculated. All analyses were performed for the total population and were stratified for trans women and trans men.

All analyses were performed using STATA Statistical software (Statacorp, College Station, TX, USA), version 15.1.

## Results

The characteristics of the study population are shown in Table 1. In total, 8263 people attended the gender identity clinic, of which 5107 were trans women (median age at first visit 28 years, range 4 to 81 years) and 3156 were trans men (median age at first visit 20 years, range 4 to 73 years). The median follow‐up time was 7.5 years (range 0.0–45.5 years), which was longer in trans women (10.2 years, range 0.0–45.5 years) than in trans men (4.8 years, range 0.0–45.5 years). The total follow‐up time was 92 227 person years (64 287 in trans women and 27 940 in trans men).

**Table 1:** Characteristics of the study population (A) and the people who died by suicide (B)

	Total	Trans women	Trans men
(A)			
Number of people	8263	5107	3156
Age at first visit, year	25 (4–81)	28 (4–81)	20 (4–73)
Follow‐up time, year	7.5 (0.0–45.5)	10.2 (0.0–45.5)	4.8 (0.0–45.5)
(B)			
Number of suicides	49 (0.6%)	41 (0.8%)	8 (0.3%)
Age at first visit, year	31 (15–59)	31 (15–58)	21 (16–59)
Age at time of suicide, year	41 (18–66)	41 (18–66)	36 (21–60)
Follow‐up time, year	6.7 (0.6–32.7)	6.7 (0.6–32.7)	6.7 (0.6–23.1)
Time between start hormones and suicide, year	6.4 (0.4–32.5) *n = 42*	6.1 (0.4–32.5) *n = 35*	6.9 (3.7–23.1) *n = 7*

Data are shown as number or median (range).

Forty‐nine people died by suicide: 41 trans women (0.8%) and 8 trans men (0.3%), which is 64 per 100 000 person years in trans women and 29 per 100 000 person years in trans men. The median follow‐up time between first visit and suicide death was 6.7 years (range 0.6 to 32.7 years) in trans women and 6.7 years (range 0.6 to 23.1 years) in trans men. Trans women had a higher overall suicide death risk than trans men (per year: HR 2.26, 95% CI 1.06–4.82). Four suicide deaths occurred in individuals who were referred to the clinic before the age of 18 (0.2%), which is a lower risk than in adults (0.7%, *P* = 0.010).

**Fig. 1:**
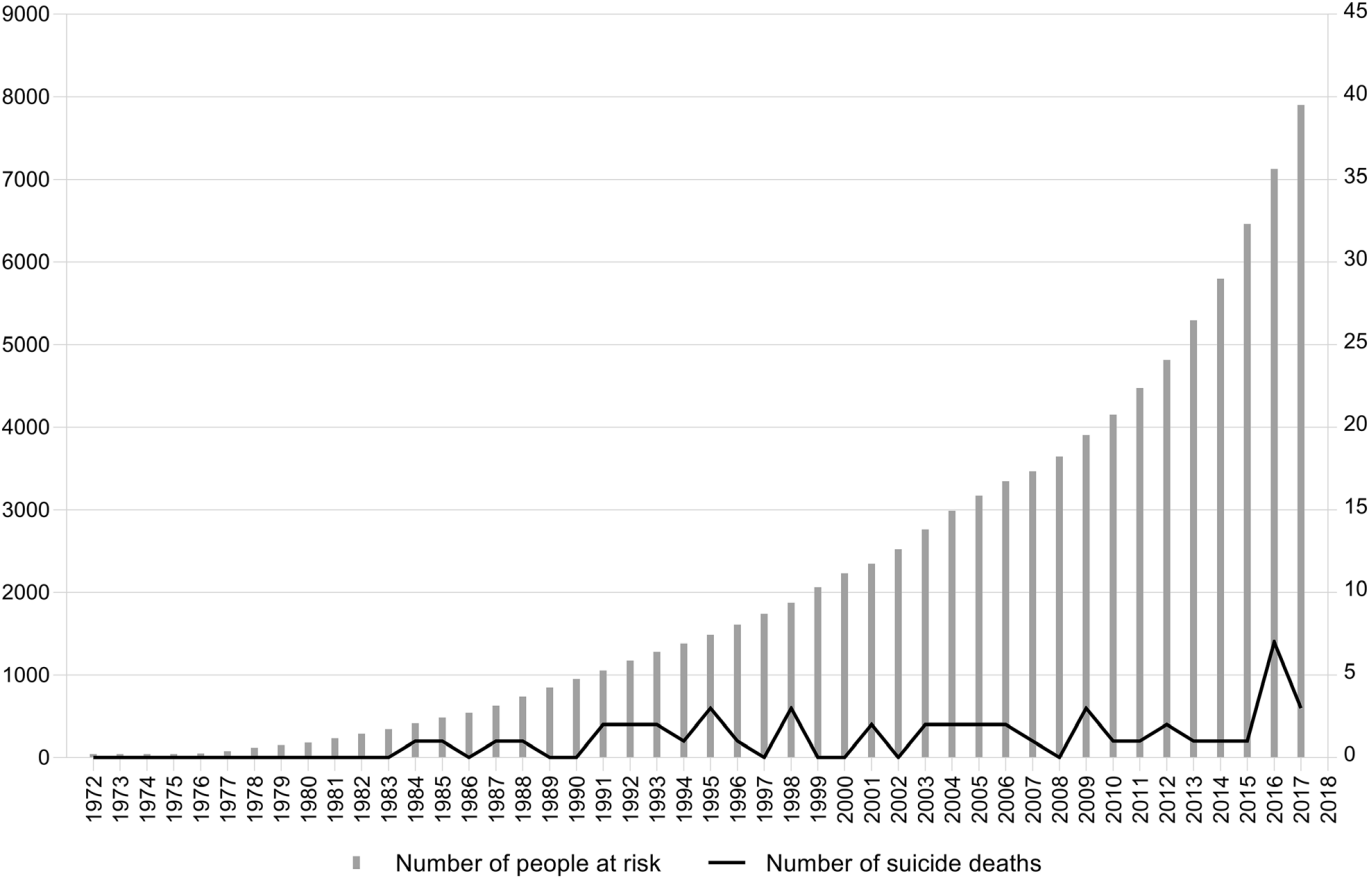
Number of people at risk (left *y*‐axis) and the number of suicides (right *y*‐axis), between 1972 and 2017.

The course of number of people at risk and the number of people who died by suicide over the years is shown in Fig. 1. Overall suicide deaths did not increase over the years: HR per year 0.97 (95% CI 0.94–1.00). In trans women, suicide death rates decreased slightly over time (per year: HR 0.96, 95% CI 0.93–0.99), while it did not change in trans men (per year: HR 1.10, 95% CI 0.97–1.25). Adjustment for age at the first visit did not change these numbers.

As the median follow‐up time between first visit and suicide death was 6.7 years, subgroup analyses were performed in those who had their first visit before 2011. This did not change the outcomes: trans women (*n* = 3115) HR 0.94, 95% CI 0.91–0.98; trans men (*n* = 1269) HR 1.02, 95% CI 0.90–1.16).

Of the 49 people who died by suicide, 35 had a face‐to‐face contact with the endocrinologist or psychologist of the gender identity clinic in the previous two years, while the other 14 people were no longer in active counseling with the clinic. Sixteen of the 35 people who recently had visited the clinic, only came for a medical check‐up, as they were postsurgery (vaginoplasty or phalloplasty). Two people were in the surgery trajectory, and 17 were still in the diagnostic or hormonal phase at time of suicide. The transition phases separately for trans women and trans men who died by suicide are shown in Table 2.

**Table 2:** The occurrence of suicide deaths distinguished for transition stage, and trans women or trans men

	Total (*n* = 49)	Trans women (*n* = 41)	Trans men (*n* = 8)
In active counseling	35	29	6
In diagnostic or hormonal phase	17	16	1
In surgical phase	2	0	2
Only medical follow‐up care	16	13	3
No active counseling	14	12	2

Data are shown as number. In active counseling is defined as a face‐to‐face contact with the endocrinologist or psychologist of the gender identity clinic in the previous two years.

The mean number of suicides in the years 2013–2017 was higher in the trans population (40 per 100 000 person years; 43 per 100 000 trans women and 34 per 100 000 trans men) compared with the Dutch population in this time frame (11 per 100 000 person years; 15 per 100 00 registered men and 7 per 100 000 registered women) (28).

## Discussion

The current study investigated the suicide death risk in the largest clinical cohort of gender‐referred individuals to the Center of Expertise on Gender Dysphoria at the Amsterdam UMC, the Netherlands, between 1972 and 2017. Findings from the chart reviews showed us a decrease in suicide death risk over time in trans women and no change in suicide death risk in trans men. Trans women, however, showed a higher suicide death risk than trans men. Between 2013 and 2017, the suicide risk in Dutch referred transgender people (40 per 100 000 person years) showed to be three to four times higher than the general Dutch population (11 per 100 000 person years) (28). Evaluation of transition stage in relation to suicide deaths showed that approximately two‐third of the observed suicides occurred in those who were still in active treatment (diagnostic, hormonal, or surgical phase). The incidence of suicide deaths and transition stage was similar in trans women and trans men.

Suicidal behaviour is a complex phenomenon that is a result of many individual (age, male sex assigned at birth, previous suicide attempts, mental health history, substance abuse) as well as more distant environmental factors. A recent literature review clearly demonstrates the specific risk factors for suicide in sexual minority youth, which includes negative social environments, inadequate support within the closest social network, and an absence of lesbian, gay, bisexual, and transgender (LGBT) movements in communities (29). In our cohort, both trans women and trans men show a three‐ to four‐fold elevated risk of suicide compared with the population rate in the Netherlands and can therefore be considered a high‐risk group. Although the Netherlands is known for its tolerance toward sexual minority groups in comparison to most countries in the world (30), the societal position of trans people is generally less favorable compared with the lesbian, gay, bisexual, and cis‐gender population. Furthermore, compared with trans men, the societal position of trans women is lower (31, 32.

In the Netherlands, between 1972 and 2017 suicide rates showed a fluctuating course. Our finding of a slightly decreasing suicide risk in Dutch trans women may confer some hope. Recent studies showed an increase in societal acceptance toward lesbian, gay, bisexual, and transgender people (31), and indications of an increase in social‐economic status over the years (33). Although specific information on trans men and trans women is unavailable, it is conceivable that the improvement of societal position may have effect on the psychological functioning and the prevention of suicidal risk in trans women. The cause of this increase in tolerance seems largely to be the effect of a national and international increase in visibility and attention for trans people in media and society. Another explanation may be that, with the increase in attention and acceptance, the threshold for transgender people to seek treatment or professional help has become lower over the years. This is also reflected by the increase in referrals each year (26). Lastly, with the increase of knowledge in this field and the literature about the vulnerability of the transgender population for suicidal ideation, suicidal attempts, and suicide death rates, it is conceivable to assume that the attention to these risks has increased in clinical counseling and may have its effect on prevention of suicide deaths over the years.

Although the incidence of suicide deaths in trans women decreased over the years, the overall incidence still showed to be higher in trans women compared with trans men. Conflicting results in literature are reported about the risk of suicide attempts between trans women and trans men. Some studies reported that trans men had a higher risk of suicide attempts than trans women (22, 23, while in other studies no differences in suicide attempts between trans women and trans men were found (24, 25. Only two studies looked at the differences in the risk of death by suicide between trans women and trans men and found that trans women had an increased risk compared with trans men (20, 21. However, these two studies were earlier studies performed in our center and therefore include a smaller part of our current study population.

An important finding was that the incidence for observed suicide deaths was almost equally distributed over the different stages of treatment. Although the distribution showed that one‐third of the suicides occurred in people who were no longer in active treatment in our center, the other two‐third of the people who died by suicide still visited our center in the previous two years. About half of these last two‐third people were still in active diagnostic or medical treatment, while the other half completed their transition and only came for a medical check‐up. This indicates that vulnerability for suicide occurs similarly in the different stages of transition. Although the literature on suicide risk factors is comprehensive, and particular suicidal risk factors like verbal victimization, physical and sexual violence, and the absence of social support (9, 34, may apply for transgender people in all stages of transitioning, it seems clinically highly relevant to understand and explore possible differences in motives and risk factors in the different stages of treatment. Therefore, future research on suicide deaths and suicide risk factors in transgender people should have a greater focus on transition status in relation to these motives and risk factors.

This study is performed in the largest cohort of gender‐referred people from the Netherlands, consisting of a large population of both adult and adolescent trans women and trans men at different stages of their transition with a long follow‐up time. However, this study has also some limitations. First, this study is a retrospective chart study. Although we used multiple strategies to obtain data about date of death, it is possible that we missed some data. Second, we did not have information about psychological comorbidities or other psychological information, such as social support. Third, we only had information about people who actually visited our gender identity clinic. Information about people on the waiting list for their first appointment was lacking.

To conclude, in our clinic we observed no increase in suicide death risk over time and even a decrease over time in suicide death risk in trans women was found. Since the suicide risk in the transgender population is higher than the general population and seems to occur during every stage of transitioning, it is important that (mental) health practitioners pay attention to this risk and create a safe environment in which these feelings can be discussed at all stages of treatment and counseling. Further research is necessary to investigate the motives behind the suicides, as input in the development of adequate suicide prevention programs.

## Conflicts of interests

None.

## Data Availability

Author elects to not share data.
